# Establishment of a high-dependency unit in Malawi

**DOI:** 10.1136/bmjgh-2020-004041

**Published:** 2020-11-19

**Authors:** Ben Morton, Ndaziona Peter Banda, Edna Nsomba, Clara Ngoliwa, Sandra Antoine, Joel Gondwe, Felix Limbani, Marc Yves Romain Henrion, James Chirombo, Tim Baker, Patrick Kamalo, Chimota Phiri, Leo Masamba, Tamara Phiri, Jane Mallewa, Henry Charles Mwandumba, Kwazizira Samson Mndolo, Stephen Gordon, Jamie Rylance

**Affiliations:** 1 Department of Clinical Sciences, Liverpool School of Tropical Medicine, Liverpool, UK; 2 Malawi-Liverpool-Wellcome Trust Clinical Research Programme, Blantyre, Malawi; 3 Department of Medicine, College of Medicine, Blantyre, Malawi; 4 Queen Elizabeth Central Hospital, Blantyre, Malawi; 5 Department of Global Public Health, Karolinska Institutet, Stockholm, Sweden

**Keywords:** treatment, cardiovascular disease, HIV, tuberculosis

## Abstract

Adults admitted to hospital with critical illness are vulnerable and at high risk of morbidity and mortality, especially in sub-Saharan African settings where resources are severely limited. As life expectancy increases, patient demographics and healthcare needs are increasingly complex and require integrated approaches. Patient outcomes could be improved by increased critical care provision that standardises healthcare delivery, provides specialist staff and enhanced patient monitoring and facilitates some treatment modalities for organ support. In Malawi, we established a new high-dependency unit within Queen Elizabeth Central Hospital, a tertiary referral centre serving the country’s Southern region. This unit was designed in partnership with managers, clinicians, nurses and patients to address their needs. In this practice piece, we describe a participatory approach to design and implement a sustainable high-dependency unit for a low-income sub-Saharan African setting. This included: prospective agreement on remit, alignment with existing services, refurbishment of a dedicated physical space, recruitment and training of specialist nurses, development of context-sensitive clinical standard operating procedures, purchase of appropriate and durable equipment and creation of digital clinical information systems. As the global COVID-19 pandemic unfolded, we accelerated unit opening in anticipation of increased clinical requirement and describe how the high-dependency unit responded to this demand.

**Summary box**

- There is an unmet need for critical care services for adults with unstable medical conditions in low-resource settings.
- We sought to establish a high-dependency unit nested within the existing healthcare system.
- We describe a prospective approach to cocreate a sustainable high-dependency unit relevant to low-resource settings.
- Opened as the COVID-19 epidemic unfolded, the high-dependency unit was instrumental to the delivery of standardised care for patients with severe respiratory and comorbid disease.

## Introduction

**Figure 1:**
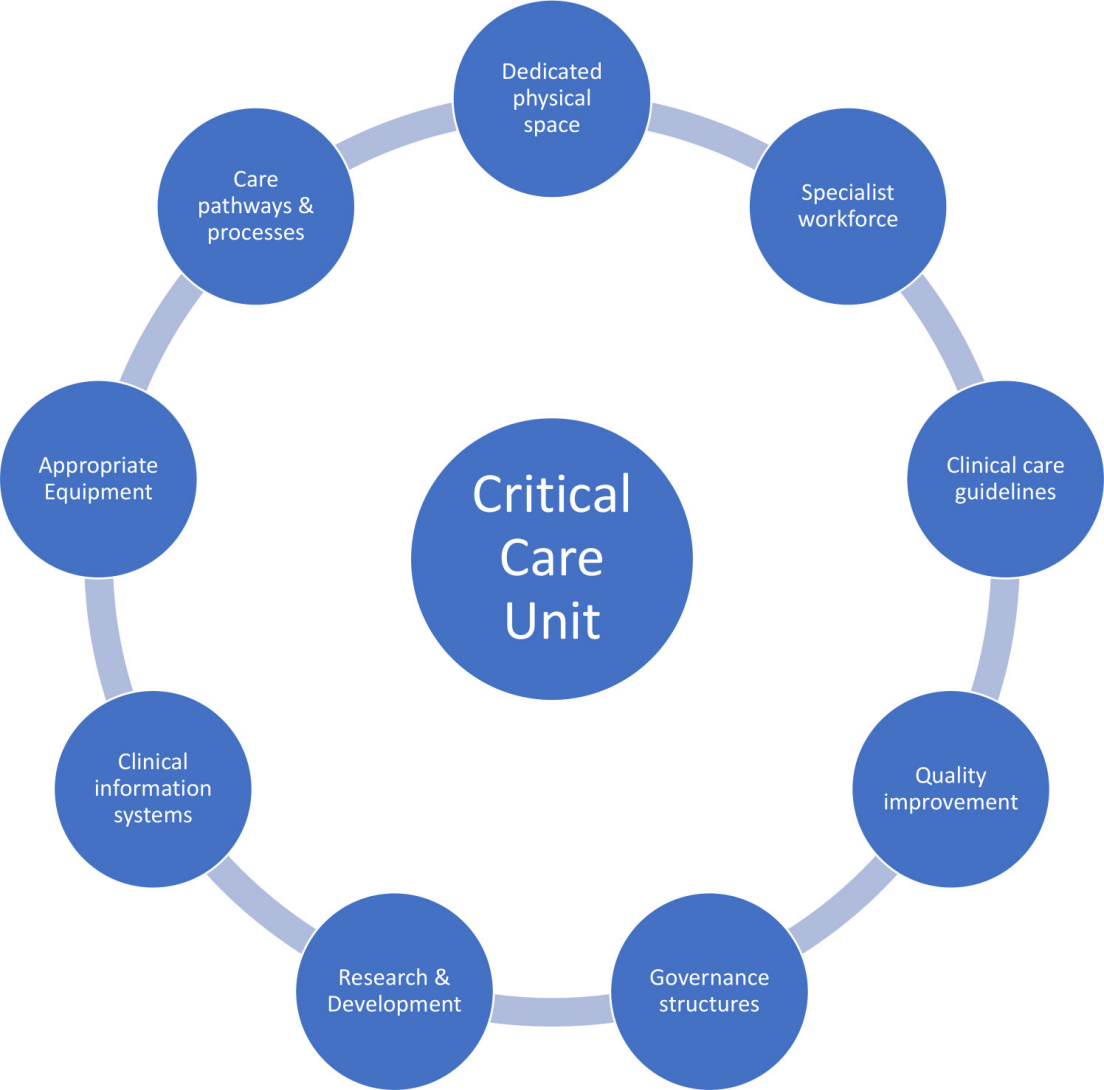
Key components for critical care delivery. Summary figure developed from the Faculty of Intensive Care Medicine UK guidelines for the provision of intensive care services.^14^

Adults with acute medical conditions in low-income sub-Saharan African countries often present late to healthcare. Patients may subsequently be faced with a lack of robust referral pathways and resources in secondary care settings. This increases vulnerability and contributes to the high observed rates of morbidity and mortality. Specialist critical care units improve survival in high-income countries (HICs)^1^ and have potential to significantly improve prognosis in low-income and middle-income countries (LMICs) where the mortality rates of critically unwell patients remain unacceptably high.^2^ Contextual differences mean that transplanting the model of intensive care directly from HIC to LMIC may not be feasible or desirable.^3^ However, provision of increased nursing care and improved monitoring for unstable critically unwell patients could profoundly improve patient outcomes. Pre-COVID-19 in Malawi, there were 16 working ventilators across four central hospitals in a survey on 16 May 2020^4^; this equates to 0.1 ‘conventional’ intensive care beds with ventilator capability per 100 000 population (compared with 27.0 per 100 000 in USA).^5^ However, thinking in terms of ‘beds’ and ‘ventilators’ distracts from the critical shortage of healthcare workers and underdeveloped healthcare pathways required to deliver critical care. Developing the workforce requires deep and consistent investment.^6^ Our aim was to support the development of a specialist medical critical care area integrated within the existing healthcare system at Queen Elizabeth Central Hospital (QECH), Blantyre. We concentrated on critically unwell medical patients as this vulnerable population was underserved within the hospital compared with surgical patients. Here, we describe our experience of the process: designing and renovating the ward space; establishing guidelines, pathways and procedures; workforce training; and developing clinical information systems. Figure 1 describes the key components for successful establishment of critical care that healthcare delivery managers should consider during planning and delivery.

## Setting

QECH is a government hospital of 1000 beds, providing free inpatient medical care to the city of Blantyre (population 800 264) and tertiary care to those referred from the Southern region (population 7 750 629).^7^ Most adults present directly to the hospital or are referred from community healthcare facilities. After triage, stabilisation and specialty referral in the dedicated emergency department, medical patients are admitted onto one of two single sex adult wards. Each ward houses between 50 and 100 patients (demand varies widely according to season) and a ward ‘high dependency area’ that accommodates up to eight unstable patients. These areas receive increased clinical supervision (twice daily consultant ward rounds and a nurse ratio of 1 to approximately 20 patients) and have access to oxygen delivered via cylinders and concentrators; these are mostly unavailable in the main ward. Physiological monitoring and nursing provision are similar throughout the wards, although efforts are made to prioritise those in the high-dependency areas. Our data show high morbidity and mortality in these high-dependency patients, commonly due to pneumonia (14.6%)^8^ and sepsis (28.1%).^9^ Malawi has seen a rapid increase in life expectancy as antiretroviral treatment programmes have rolled out from a mean age of 45.6 in 2000 to a projected 64.3 in 2020.^10^ Trends in QECH medical admissions reflect this with a decreasing proportion of HIV-infection positive patients and an increasing proportion of older, HIV-infection negative patients with non-communicable disease (eg, hypertension and diabetes) complications.^11^ This rapidly changing population presents to hospital with increasingly complex multimorbid disease requiring more specialised and integrated care.

A separate four-bed adult intensive care unit is overseen by the anaesthetic department. This unit has four operational ventilators and primarily cares for patient with trauma and in the postoperative period. However, patients with medical disease complications are not prioritised for intensive care unit admission. Instead, the unit is primarily used for postoperative monitoring and stabilisation for less complex patients immediately after surgery. An ongoing qualitative study (Malawi College of Medicine Research Ethics Committee approval P.03/19/2625) sought perspectives of care quality among patients with sepsis, their caregivers and healthcare workers (HCWs). This was indicative of experiences of wider acute and critical illness at the hospital and informed our high-dependency unit (HDU) design and development. Patients and HCWs raised significant issues, including an overwhelming workload for HCW, limited time with clinicians, high patient density on the wards and lack of equipment such as access to oxygen and suction machines. Despite having care delivered in such a challenging environment, patients felt that HCWs were generally caring and improvised in the face of limited resources to help patients recover.

They gave us a bed, but the ward was very full. The beds were close to one another. If you tried to put your legs on the floor, you would be touching another bed. You wouldn’t know what your neighbour is suffering from, so it is much of a risk, you can get new infections.

***In-depth interview quote from a female patient with sepsis recruited to a qualitative study that explored perspectives of care quality.***

These extreme resource constraints prompted the creation of a dedicated and sustainable HDU to improve care for this vulnerable and underserved population of medical patients. The unit was planned in collaboration between QECH and the Malawi-Liverpool Wellcome Trust Clinical Research Programme (MLW), a research centre affiliated with the Malawi College of Medicine and situated adjacent to QECH. Preventing death from severe infection is a major programmatic theme for MLW.^12^ The Wellcome Trust recognised the unmet need for adult medical hospital admissions with critical illness during the MLW core grant renewal process and provided specific funding to create the unit.

## HDU remit

Critical care has been defined by its key components: a distinct physical space, organised systems for care provision, specialist staff, enhanced patient monitoring and multiple modalities for providing organ support.^13^ In the UK, four levels of hospital care are recognised (table 1)^14^

**Table 1:** Levels of critical care

Level	Definition
0	Patients whose needs can be met through normal ward care in an acute hospital.
1	Patients at risk of clinical deterioration, or those recently relocated from higher levels of care, whose needs can be met on an acute ward with additional specialist advice and support from the critical care team.
2	Patients requiring more detailed observation or intervention including support for a single failing organ system, those receiving postoperative care or those ‘stepping down’ from level 3 care.
3	Patients requiring advanced (ventilatory) respiratory support alone or basic respiratory support together with support of two or more organ systems. This level includes all complex patients requiring support for multiorgan failure.

Prior to commencement of the project, preliminary round table discussions between institutional leaders developed a shared vision of the project. This culminated in a memorandum of understanding between the hospital and MLW directors, leading to regular meetings of key medical, nursing and research staff (‘the team’) to operationalise the vision. We aimed to provide an HDU that addressed limitations in level 1 and 2 care provision for medical patients. Three key areas of need were identified: (1) a dedicated physical space, designed specifically for the care of critically unwell patients; (2) provision of specialist critical illness nurses at increased ratio compared with standard ward care; and (3) uninterrupted oxygen supplementary supply. With this foundation, we expected that the unit would be sustainable, responsive to future needs and realistic given a limited, fixed budget. We held weekly meetings with a wide group of stakeholders (including the clinical and nursing leadership, facilities and estates, and service users) to prospectively develop a shared understanding of what services would be offered. These meetings were vital to manage expectations of the available resources and project scope, including prospective discussions around provision of advanced functions such as ventilation and arterial blood gas monitoring. Through discussion, the team agreed that provision of high-quality nursing and monitoring care were the strategic focus based on the potential for sustainable benefit to patients. The team included plans for high-fidelity data collection to facilitate analysis of patient outcomes and engender a culture of continuous quality improvement.

## Infrastructural refurbishment

**Figure 2:**
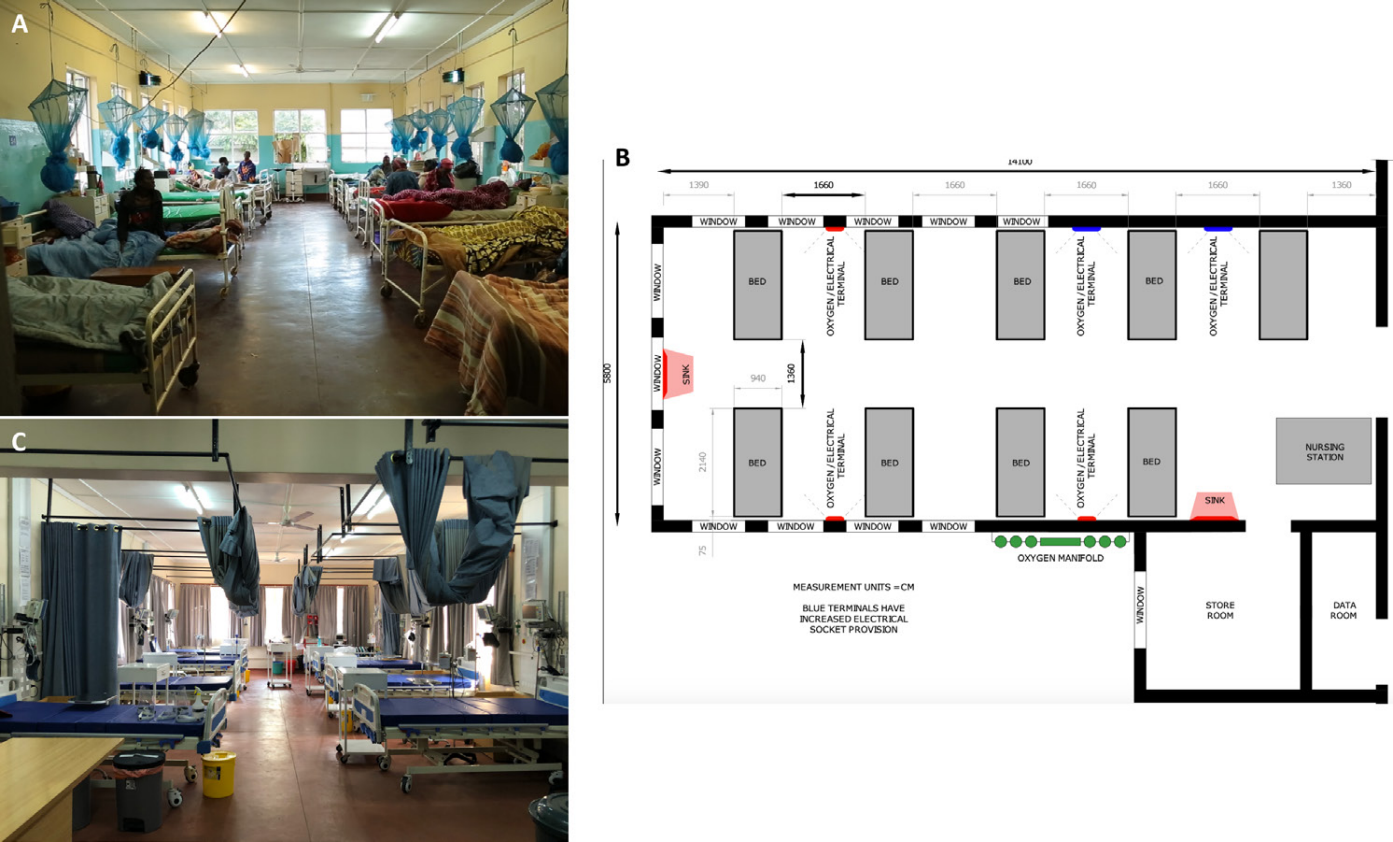
Infrastructural refurbishment. (A) Ward prerefurbishment; (B) floor plan; and (C) ward postrefurbishment. Note: Blantyre is situated 1039 m above sea level and has very low vector density and disease transmission within the city. Therefore, mosquito nets were not replaced during the refurbishment.

The hospital leadership identified, and made available, a ward area for refurbishment. Availability of separate space had resulted from changes to tuberculosis treatment regimens that reduced inpatient numbers. Geographically, this space was directly adjacent to the male medical ward and convenient for referrals and step downs. MLW recruited a senior nurse, experienced in critical care service development (EN); a consultant in critical care medicine (BM); and a construction project manager (SA) to deliver refurbishment activities. Progress updates were provided at the weekly meetings and specific ‘task and finish’ objectives agreed with stakeholders. This triumvirate approach ensured that clinical needs informed the construction process from the outset. Our space constraints allowed an optimal configuration as a nine-bed unit, incorporating a nursing station and hand washing facilities (figure 2). Given financial constraints, the allocated space was the most important determinant of the unit size. Some staff were uncomfortable that the relatively small number of beds would severely constrain the medical department’s capacity for critical care. Recognising these concerns fed highly productive discussions about patient selection, targeting of resources and efficient referral and discharge pathways.

The multidisciplinary approach to ward design drove explicit prioritisation of our unit’s function and helped crystallise important teaching points among the clinical team. For example, the plan to position the sickest patients closest to the nursing station prompted careful thought on spacing and the configuration of electrical sockets to support the increased monitoring and treatment modalities (eg, provision of infusion pumps) that might be required. Coordination through the project manager linked these service needs to infrastructural reality: the building of medical gas pipelines and safe storage areas, installation of electrical manifolds and hardware for data connections and laying of an appropriate cleanable flooring. As the COVID-19 pandemic began to unfold in early 2020, this process was accelerated in anticipation of increased clinical demand. Works were completed in March 2020, before the first cases were detected in Malawi, and the unit handed over to clinical staff for final preparations.

## Nurse recruitment and training

High workload and low staff to patient ratios are associated with poorer outcomes for patients with critical illness.^15^ The team determined to aim for ratios of not more than four patients per nurse on the HDU, necessitating a team of 10 nurses and 4 healthcare assistants. Registered nurses were recruited in August 2019, with five employed by MLW and five employed by the hospital under the leadership of EN (MLW) and her deputy (CN, QECH). Prior to unit opening, nurses rotated through critical care areas within the hospital. The nurses found this extremely useful in providing a strong grounding in critical care approaches. In addition to experiential learning, we designed and delivered bespoke training that specifically aimed to empower the nursing cadres and give confidence in their new roles. Foundational training included completion and interpretation of observation charts, and governance and data collection activities. Nurses also identified more advanced learning needs, resulting in a tailored approach.

After completion by the construction team, nurses were deployed on the newly refurbished HDU and undertook 10 days of preparatory work and orientation activities including in situ simulation training prior to acceptance of patient admissions. Initially, two beds were opened while clinical processes were troubleshooted before the entire bed base was made available to admissions. Continuous training was provided after the unit opened, focused on topics that were responsive to real-world clinical problems encountered during opening, and core competencies. Specific examples included enhanced training on patient monitors, safe titration of oxygen guided by patient SpO_2_, management of poorly controlled diabetes and prone-positioning techniques in anticipation of patients requiring admission for severe COVID-19 infection.^16^

## Clinical standard operating procedures

In parallel with nurse training, the team developed standard operating procedures (SOPs) to govern HDU activities. These were developed in partnership with clinical and nursing teams to ensure standardisation across the existing hospital systems. We specifically engaged with emergency department, intensive care and medical practitioners to ensure that expectations of unit capabilities were aligned. In parallel, governance and condition-specific SOPs were drafted by EN, JR and BM with specialist input where required. These SOPs were deliberately context sensitive and developed collaboratively with partners in the existing healthcare system to ensure that aims and objective were deliverable and sustainable. SOPs were made widely available to healthcare workers in printed and electronic formats and used as base training material for nursing staff working on the unit. We anticipated that clinical need for high dependency care would outweigh resource availability, so we prospectively agreed on admission criteria to focus on acute and treatable medical conditions.

The team agreed on criteria for those patients who may not be suitable for HDU admission including: terminal medical conditions more appropriately treated with palliative care, conditions with no available definitive treatment in Malawi, conditions requiring specific treatments outside the scope of the unit and a reduced or deteriorating conscious level caused by medically irreversible cerebral damage (eg, massive cerebrovascular accident). We also agreed that the unit should aim to operate below full capacity to ensure that a bed is readily available for rapid patient admission and stabilisation. There is evidence that a critical care bed occupancy rate of 70%–75% is optimal for critical care delivery and patient outcome.^17^ In addition to our admission criteria, the team also agreed with stakeholders that recovering patients, stabilised after acute illness, would be given priority for step down to ward level care to maintain unit capacity.

## Equipment

Through the clinical leadership team (EN, BM, JR and NB), we prospectively formulated a list of equipment mapped to the intended capabilities of the unit. We prioritised this list into essential and preferred items and began the procurement early in the refurbishment process (November 2019). Equipment prioritisation and procurement for low-income countries is challenging. Existing evidence demonstrates that 40%–70% of medical devices and equipment in LMICs are broken, unused or unfit for purpose.^18^ To reduce this risk, we prospectively engaged with colleagues from the adult and paediatric ICUs to ensure that procured items were maximally compatible with existing equipment and to learn from local experience. Essential to this process was partnership with the hospital maintenance department to ensure that all the equipment purchased was serviceable on-site. Through this engagement, we were able to access an existing global north–south knowledge exchange partnership within the hospital^19^ to facilitate purchase of durable patient monitors with skilled hospital engineers. Engineers led on equipment training to promote patient safety and device durability. Training was delivered using dedicated supervised in situ simulation training with the equipment prior to unit opening. This method enabled systems testing within a controlled environment before the unit was opened to patients.^20^

## Information systems

Building on an existing electronic hospital registry system,^21^ the team decided to prospectively capture demographic and clinical outcome data for patients admitted to the HDU. We determined that high-quality and accessible data would facilitate a continual cycle of audit and quality improvement activities. Based on experience from the Intensive Care National Audit & Research Centre (ICNARC)^22^ and observing low-income and middle- income critical care registry work from other groups,^23^ a data specialist (JG) and clinician (BM) adapted the ICNARC data collection tool for the Malawi setting. All patients admitted to the hospital have a unique identifier attached to their health passport (a universally used patient-held written health record). This existing electronic system collects basic demographic and outcome data and informs knowledge of local epidemiology.^9^ We used open source (Open Data Kit (ODK)) electronic software on a tablet device to prospectively collect daily admission, follow-up and discharge data for all patients admitted to the unit. Anonymised data were linked using the existing unique identifier to determine outcomes at high dependency and hospital discharge (online supplemental figure S1). MLW data management systems and servers adhere to globally recognised relevant standards to protect patient information, including ICH-GCP, GCDMP, GDPR, CDISC/CDASH and relevant Malawi data protection legislation. We piloted and refined the data collection tool with nursing staff before roll-out on the unit in April 2020. A data dashboard report was codesigned by the data and statistics departments using open source R statistical software. Data are collated and disseminated to nursing and clinical teams on a monthly basis, although the approach has potential for real-time feedback (see online supplemental figure S1). The ‘R’ package and ODK data dictionary are freely available on GitHub (https://github.com/mlw-stats/HDRU). At the time of writing, implementation of our planned monthly morbidity and mortality meetings to review these data have been delayed due to the COVID-19 epidemic.

10.1136/bmjgh-2020-004041.supp1 Supplementary data

## The COVID-19 experience

The first case of COVID-19 infection was confirmed in Malawi on 2 April 2020.^24^ Blantyre subsequently became a focus of the national epidemic, and multiple patients required admission to QECH for oxygen support. In response, hospital services were reorganised, and the HDU was incorporated within the COVID-19 ‘hot’ area to cohort and manage suspected and confirmed cases. The value of the HDU in providing a secure environment for clinical training and personal protective equipment provision was immediately obvious. Patients with severe hypoxia were preferentially admitted to the HDU for uninterrupted oxygen and continuous monitoring capabilities. The HDU senior team supported the development of standardised evidence-based clinical guidelines to optimise patient outcomes^25^ on the unit, including provision of titrated oxygen, dexamethasone and prophylactic heparin. Clinicians were able to directly refer unstable and complex patients to the HDU for increased care provision, severely hypoxic patients were prioritised and those with steroid-induced poor blood sugar control. HDU nursing staff cascaded training on safe oxygen titration and were able to support ward areas with provision of oxygen mask interfaces (eg, 15 L non-rebreather masks). On stabilisation and titration of oxygen, patients were stepped down to the main ward prior to discharge. In parallel with direct care provision on the HDU, MLW was also able to support the effort against COVID-19 through installation of an oxygen plant supporting the entire hospital and personal protective equipment to support frontline government workers.^26^

## Conclusion

We established a bespoke HDU cocreated in partnership with local managers, clinicians and nurses to improve care delivery for vulnerable adult patients with critical illness. As the HDU matures, we plan to use our high-quality data collection to drive audit and quality improvement activities to continue improved care. This will be delivered through strong engagement with the medical department with data freely accessible to local practitioners for use. Undergraduate and postgraduate training will be offered on the unit for clinicians, nurses and multidisciplinary team members including physiotherapists and pharmacists. We will make our data collection tools available to critical care areas across the hospital and align these with electronic data capture systems already in place and in development. We will also seek to join international critical care registry networks to share learning from other LMIC contexts to improve patient care. All nurses employed on the unit perform a hybrid role of clinical care and research delivery. We plan to use this unique resource to conduct translational research to better inform care for patients with severe illness. This clinical/research model will be used to secure sustainable funding to maintain and advance HDU care capabilities for patient benefit. We plan qualitative work to determine if and how the unit has influenced patient perspectives of acute care quality.
