# Supplementary material for: Establishment of a high-dependency unit in Malawi
Source: BMJ Glob Health. 2020 Nov 19;5(11):e004041. doi: 10.1136/bmjgh-2020-004041 (PMC7678231; doi:10.1136/bmjgh-2020-004041)
Supplement: Supplementary data [file bmjgh-2020-004041supp001.pdf]

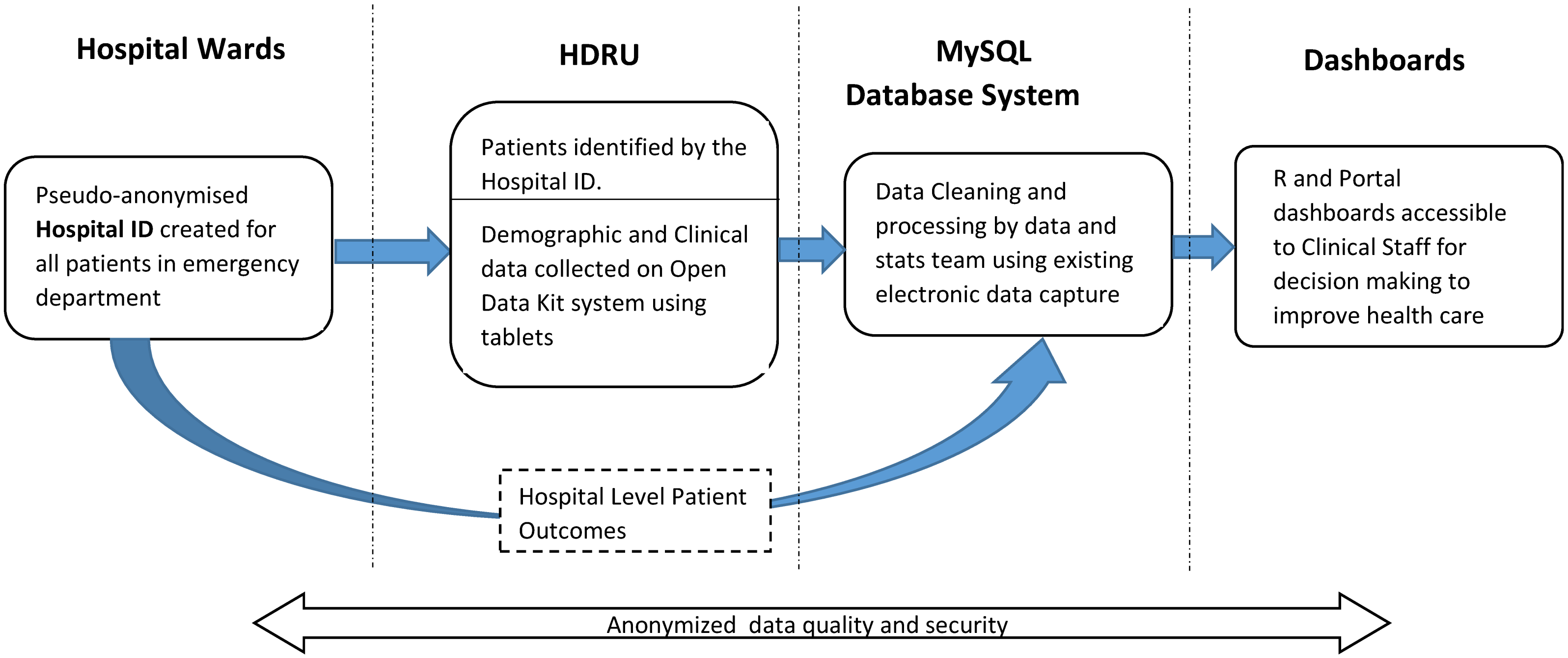

Figure S1: Data flow chart demonstrating HDRU data handling. Our data collection tool is nested within an existing hospital admission electronic registry system that assigns a unique identifier for every patient admitted to hospital. This existing system records basic demographic data and hospital outcome data. Using the same identifier, the HDRU data collection tool supplements this data set with HDRU relevant clinical variables.
